# Oleate-induced PTX3 promotes head and neck squamous cell carcinoma metastasis through the up-regulation of vimentin

**DOI:** 10.18632/oncotarget.17326

**Published:** 2017-04-21

**Authors:** Shih-Hung Chan, Jhih-Peng Tsai, Chih-Jie Shen, Yu-Han Liao, Ben-Kuen Chen

**Affiliations:** ^1^ Division of Cardiology, Department of Internal Medicine, National Cheng Kung University Hospital, College of Medicine, National Cheng Kung University, Tainan 701, Taiwan, People's Republic of China; ^2^ Institute of Bioinformatics and Biosignal Transduction, College of Bioscience and Biotechnology, National Cheng Kung University, Tainan 701, Taiwan, People's Republic of China; ^3^ Graduate Institute of Medical Sciences, College of Medicine, Taipei Medical University, Taipei 110, Taiwan, People's Republic of China; ^4^ Department of Pharmacology, College of Medicine, National Cheng Kung University, Tainan 701, Taiwan, People's Republic of China; ^5^ Department of Biotechnology and Bioindustry Sciences, College of Bioscience and Biotechnology, National Cheng Kung University, Tainan 701, Taiwan, People's Republic of China; ^6^ Institute for Cancer Biology and Drug Discovery, College of Medical Science and Technology, Taipei Medical University, Taipei 110, Taiwan, People's Republic of China

**Keywords:** oleate, PTX3, metastasis

## Abstract

The association between metabolic diseases and the risk of developing cancer is emerging. However, the impact of long pentraxin-3 (PTX3) on dyslipidemia-associated tumor metastasis remains unknown. In this study, we found that oleate induced PTX3 expression and secretion through the activation of Akt/NF-κB pathway in head and neck squamous cell carcinomas (HNSCCs). The activation of NF-κB was essential for the oleate-induced stabilization of PTX3 mRNA. In addition, both the depletion of PTX3 and the inhibition of NF-κB significantly inhibited oleate-induced tumor cell migration and invasion. The enhancement of binding between tumor and endothelial cells was observed in oleate-treated cells but not in the depletion and neutralization of PTX3 with siPTX3 and anti-PTX3 antibodies, respectively. The levels of oleate-induced epithelial-mesenchymal transition (EMT) markers, such as vimentin and MMP-3, were significantly reduced in PTX3-depleted cells. Knocking down vimentin also repressed oleate-induced HNSCC invasion. Furthermore, the depletion of PTX3 blocked the oleate-primed metastatic seeding of tumor cells in the lungs. These results demonstrate that oleate enhances HNSCC metastasis through the PTX3/vimentin signaling axes. The inhibition of PTX3 could be a potential strategy for the treatment of dyslipidemia-mediated HNSCC metastasis.

## INTRODUCTION

Head and neck squamous cell carcinoma (HNSCC) is one of the most common types of cancer. Head and neck cancers are of squamous cell histology and originate in the oral cavity, tongue, lip, gum, oropharynx, nasopharynx and hypopharynx [1]. Over the past few decades, although many studies have focused on the treatment of HNSCC, the mortality rate of HNSCC has not significantly changed [2]. Metastasis is the primary contributor to the overall mortality of cancer patients. The pathogenesis of cancer metastasis involves a series of steps, including the loss of cellular adhesion, an increase in cell invasion, cell survival in the circulation during extravasation, and the eventual colonization of distant organs [3].

The association between metabolic syndrome and the risk of developing cancer is emerging. The process involves the derangement of the circulating levels of biologic mediators that are linked with metabolic syndrome and has been implicated in promoting cancer progression [4, 5]. For example, obesity affects circulating lipid levels and increases the risk of cancer [6, 7]. Cancer cells exhibit significant metabolic alterations, including increased lipid production, which is critical for cancer cell survival. In an animal study, a high-fat diet activates the self-renewal of intestinal stem cells and endows an organoid-initiating capacity to non-stem-cell progenitors by activating Peroxisome proliferator-activated receptor-δ signaling to form tumors [8]. Fatty acid synthase, the lipogenic enzyme responsible for the endogenous synthesis of fatty acids, is strongly correlated with cancer progression and has been associated with poor prognosis of HNSCC [9–11]. Moreover, obesity is an adverse prognostic variable in patients with SCC of the oral tongue [12]. On the other hand, cancer cells may take up fatty acids from circulation and diet-derived lipoprotein particles by expressing lipoprotein lipase for survival [13, 14]. In addition, adipocyte-derived lipids promote the progression of ovarian cancer metastases to the omentum [15]. The results indicate that the secreted lipids may confer metastatic abilities to tumor cells.

Chronic low-grade inflammation plays an important role in the pathogenesis of metabolic diseases [16]. Among the inflammatory proteins, long pentraxin-3 (PTX3) is produced by resident or innate immune cells in peripheral tissues in response to inflammatory signals and toll-like receptor activation [17]. The physiological roles of PTX3 in innate immunity and inflammation have been reported [17, 18]. PTX3 binds specific pathogens to promote phagocytosis and the consequent clearance of the pathogen [19, 20]. Overexpression of PTX3 enhances the inflammatory response in transgenic mice [21]. In addition, inflammation-associated diseases, including cardiovascular disease and cancer, are correlated with the expression of PTX3. A higher level of PTX3 is associated with an increased risk of myocardial infarction [22]. Evidence suggests that PTX3 serves as a biomarker of atherosclerosis and metabolic disorders [23, 24]. Furthermore, PTX3 is a serum biomarker for lung cancer and is significantly overexpressed in prostate tumor tissue [25, 26]. The activation of epidermal growth factor receptor signaling triggers the expression of PTX3, which promotes HNSCC metastasis [27]. In addition, PTX3 is a CCAAT/Enhancer Binding Protein Delta -responsive gene and serves a protumor role in breast cancer cells upon anticancer drug treatment [28]. Recent evidence has suggested that the positive expression of matrix metalloproteinase-2 (MMP-2) and matrix metalloproteinase-9 (MMP-9) was associated with PTX3 expression in the regulation of human cervical cancer cell metastasis [29]. Although the functional role of PTX3 in tumor progression and metabolic disorders has been demonstrated, the impact of PTX3 on dyslipidemia-associated tumor metastasis remains unknown.

In an effort to identify markers associated with cancer progression [30, 31], it is important to assess the role of dyslipidemia in the regulation of cancer metastasis. Because of the different clinical combinations of the various metabolic abnormalities in cancer patients, further studies of the mechanisms involved in coordinating between these metabolic disorders and cancer metastasis are urgently required. We found that increased oleate levels are associated with HNSCC-endothelial cell interactions and tumor metastasis but not tumor cell proliferation through the induction of the inflammatory compound PTX3. Oleate-induced PTX3 enhanced the expression of vimentin and matrix metalloproteinase-3 (MMP-3) to regulate tumor invasion. This observation represents a missing link in the connection between fatty acids and cancer metastasis by providing evidence that an inflammatory protein can mediate dyslipidemia-associated tumor metastasis.

## RESULTS

### Oleate induces PTX3 expression in HNSCC cell lines

**Figure 1:**
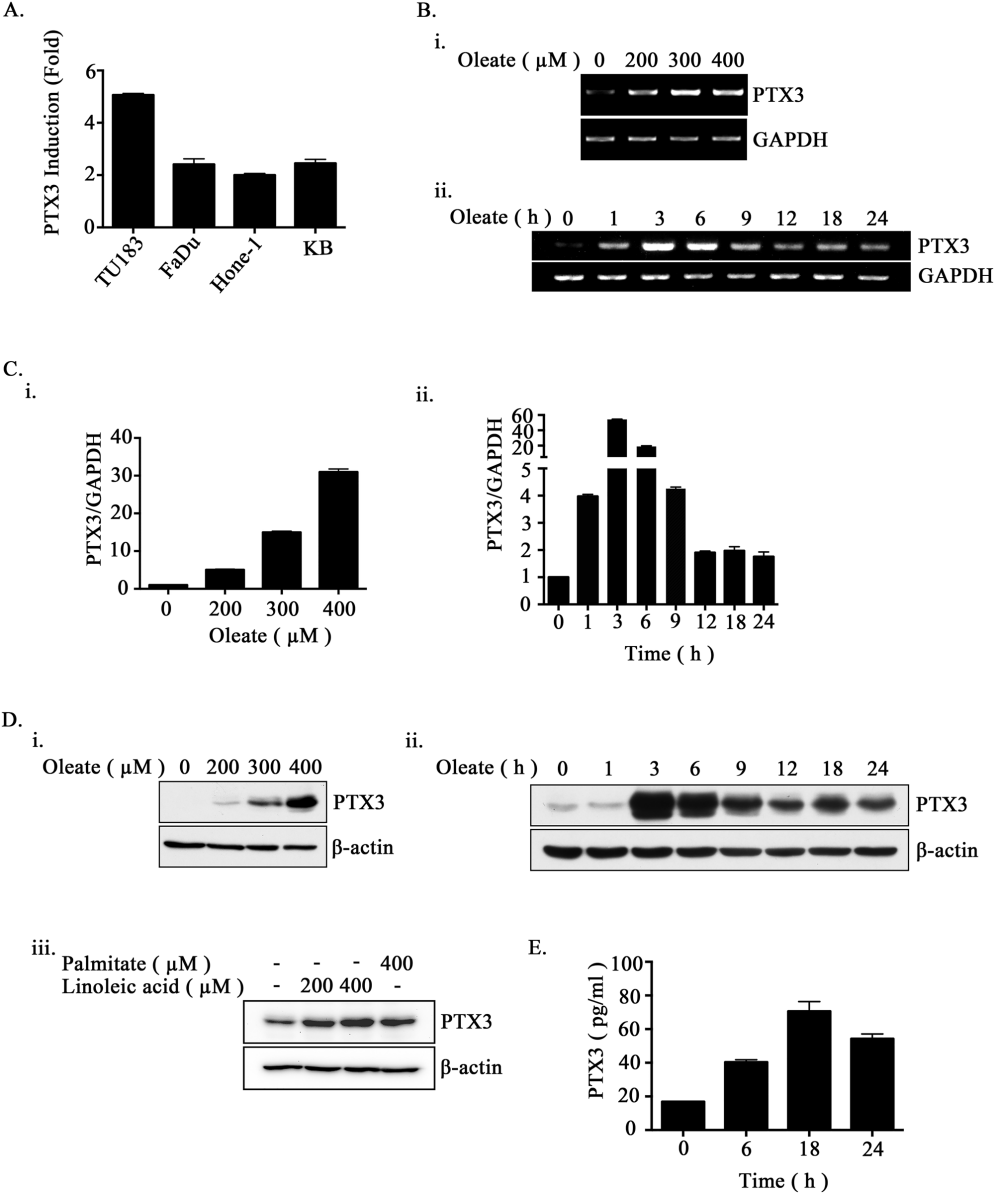
Oleate induces the expression of PTX3 in HNSCC cells **(A)** The head and neck cancer cell lines TU183, KB, FaDu and HONE1 were treated with 200 μM oleate for 6 h. The mRNA expression of PTX3 was normalized to the GAPDH mRNA level by real-time quantitative PCR. **(B-D)** TU183 cells were treated with varying concentrations of oleate, linoleic acid and palmitate for 6 h or varying periods of time as indicated. The expression levels of PTX3 mRNA were analyzed by RT-PCR **(B)** and quantitative PCR **(C)**. The protein levels in cell lysates were determined by Western blotting with antibodies against PTX3 and β-actin **(D)**. **(E)** The conditioned media from 400 μM oleate-treated TU183 cells was collected to analyze the protein levels of PTX3 by ELISA. The values represent the mean ± s.e.m. of three determinations.

PTX3 is not only an inflammatory marker but also affects lipid accumulation in human macrophages to increase oxidized low-density lipoprotein (oxLDL) uptake [32]. However, the correlation between lipid metabolite-triggered tumor metastasis and PTX3 expression remains unclear. To elucidate the relationship between lipid disorders and the expression of PTX3 in HNSCC, the effect of oleate on PTX3 expression in HNSCC cell lines was examined. Quantitative reverse transcription PCR demonstrated that PTX3 was induced in oleate-treated cells (Figure 1A). In addition, oleate induced PTX3 mRNA and protein expression in a dose- and time-dependent manner (Figure 1B-1D). In addition, palmitate and linoleic acid also significantly induced PTX3 expression (Figure 1D). To further study whether oleate promotes the autocrine production of PTX3 in tumor cells, an enzyme-linked immunosorbent assay was performed. As shown in Figure 1E, oleate significantly increased the secretion of PTX3 protein into the culture medium. These results suggest that oleate promoted PTX3 expression in the HNSCC cells.

### Oleate induces PTX3 expression through the AKT/NF-κB pathway

**Figure 2:**
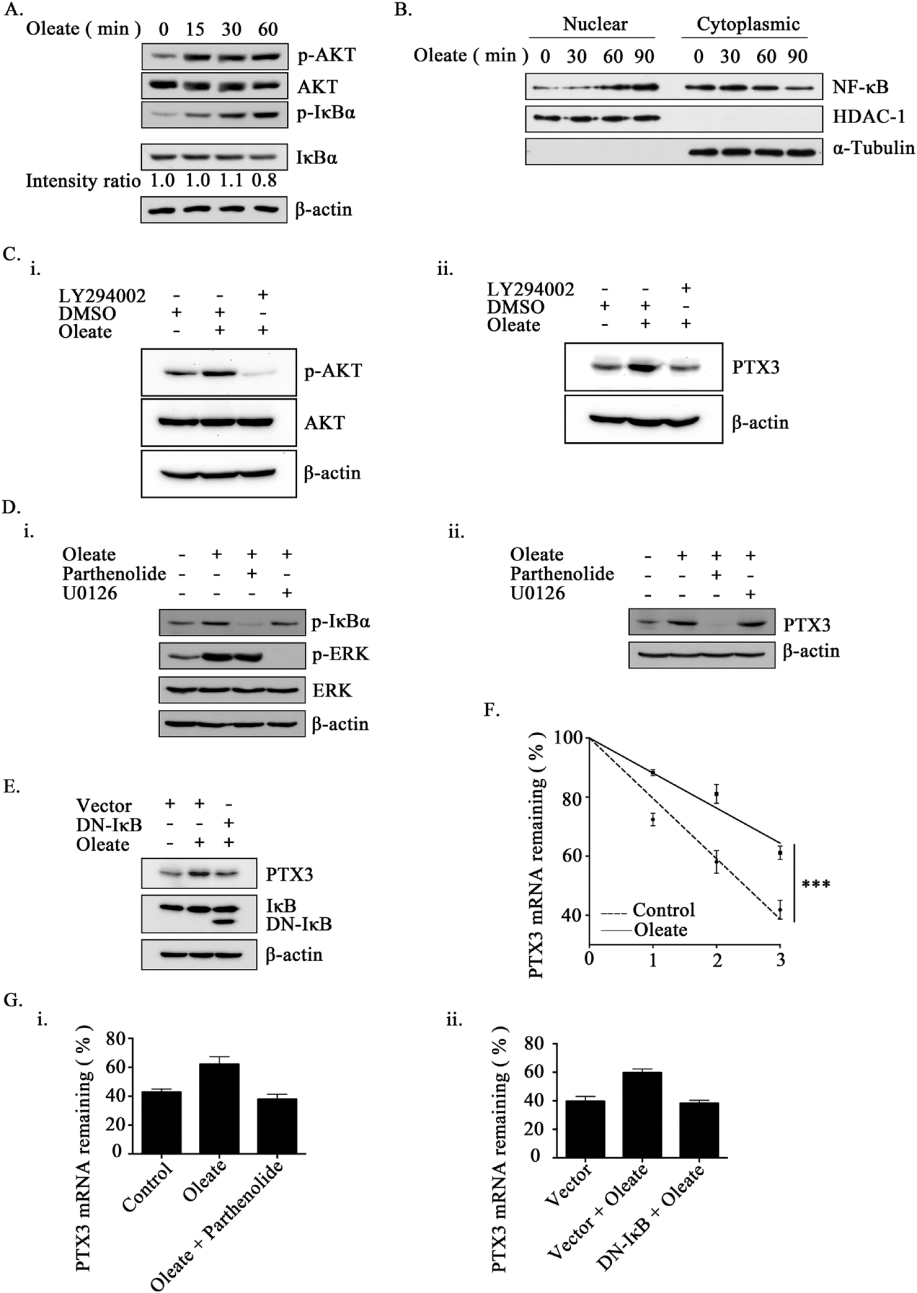
The activation of NF-κB is essential for oleate-induced PTX3 expression **(A and B)** TU183 cells were treated with 400 μM oleate for the indicated period of time. The protein levels were determined by Western blotting with antibodies against AKT, IκBα, β-actin and phosphorylated AKT and IκBα. The expression of IkBα was calculated by image-based computational quantification **(A)**. The cytoplasmic fractions and nuclear extracts of the cells were prepared in the same volume of buffer, and an aliquot of each fraction was used for Western blotting analysis using antibodies against NF-κB, HDAC-1 and α-Tubulin **(B)**. **(C)** Cells were pretreated with 20 μM LY294002 (Sigma-Aldrich, St Louis, MO, USA) for 1 h, then with 400 μM oleate for 1 h (i) or 6 h (ii). The protein levels in cell lysates were determined by Western blotting with antibodies against AKT, PTX3, phosphorylated AKT and β-actin. **(D and E)** Cells were transfected with the DN-IκB expression vector by lipofection or pretreated with 10 μM parthenolide (Sigma-Aldrich, St Louis, MO, USA) or 5 μM U0126 (Sigma-Aldrich, St Louis, MO, USA) for 1 h, then with 400 μM oleate for 1 h (i) or 6 h (ii) **(D)**. The protein levels in cell lysates were determined by Western blotting with antibodies against ERK, PTX3, IκBα, β-actin and phosphorylated IκBα and ERK. **(F and G)** Cells were treated with 400 μM oleate for 1 h and then treated with 4 μM actinomycin D (Sigma-Aldrich, St Louis, MO, USA) for the indicated period of time **(F)**. On the other hand, cells were transfected with the DN-IκB expression vector by lipofection or treated with 10 μM parthenolide and 400 μM oleate for 1 h, and then treated with 4 μM actinomycin D for 3 h **(G)**. The expression of PTX3 mRNA was analyzed by real-time quantitative PCR. Relative levels of PTX3 were normalized by GAPDH and the degradation rate was calculated by comparing to 0 h (considered as 100%). The values represent the mean ± s.e.m. of three determinations.

Our previous studies have shown that epidermal growth factor (EGF)-induced PTX3 expression was achieved through the NF-κB pathway [27]. Therefore, we hypothesized that the activation of the NF-κB pathway is essential for the regulation of oleate-induced PTX3 expression. First, we examined whether oleate activates NF-κB signaling. As shown in Figure 2A, oleate not only induced the phosphorylation of AKT but also IκBα, and it induced a decrease in IκBα protein, thus suggesting the activation of NF-κB in oleate-treated cells. Indeed, oleate enhanced the translocation of NF-κB from the cytoplasm into the nucleus in a time-dependent manner (Figure 2B and Supplementary Figure 1A). To study whether the activation of the AKT/NF-κB pathway was essential for oleate-induced PTX3 expression, inhibitors of AKT and NF-κB were used. Although the oleate-activated AKT, IκBα and ERK were inhibited by LY294002, parthenolide and U0126, respectively, only LY294002 and parthenolide significantly inhibited the oleate-induced PTX3 expression (Figure 2C and 2D). To further confirm the effect of permanently inhibiting NF-κB activation in the induction of PTX3 expression by oleate, we utilized a dominant negative form of IκB (DN-IκB) that lacked all N-terminal phosphorylation sites; thus, it is resistant to degradation but still had the ability to bind to NF-κB [33, 34]. Consistently, the oleate-induced PTX3 protein expression was dramatically reduced in the DN-IκB-expressing cells (Figure 2E). These results suggest that the oleate-induced PTX3 expression was dependent on the activation of the NF-κB pathway. To clarify whether oleate induced PTX3 expression through the activation of transcriptional regulation or the stabilization of mRNA, the effects of oleate on PTX3 promoter activity and mRNA degradation were examined. Although EGF induced the PTX3 promoter activity that was the same as our previous report [27], the luciferase reporter assay indicated that oleate had no effect on PTX3 promoter activity (Supplementary Figure 1B). However, oleate stabilized the PTX3 mRNA, but the stabilization was reduced in parthenolide-treated cells and DN-IκB expressing cells (Figure 2F and 2G). These results revealed that the activation of NF-κB by oleate was essential for the stabilization of PTX3 mRNA.

### The expression of PTX3 is essential for oleate-enhanced HNSCC migration and invasion

**Figure 3:**
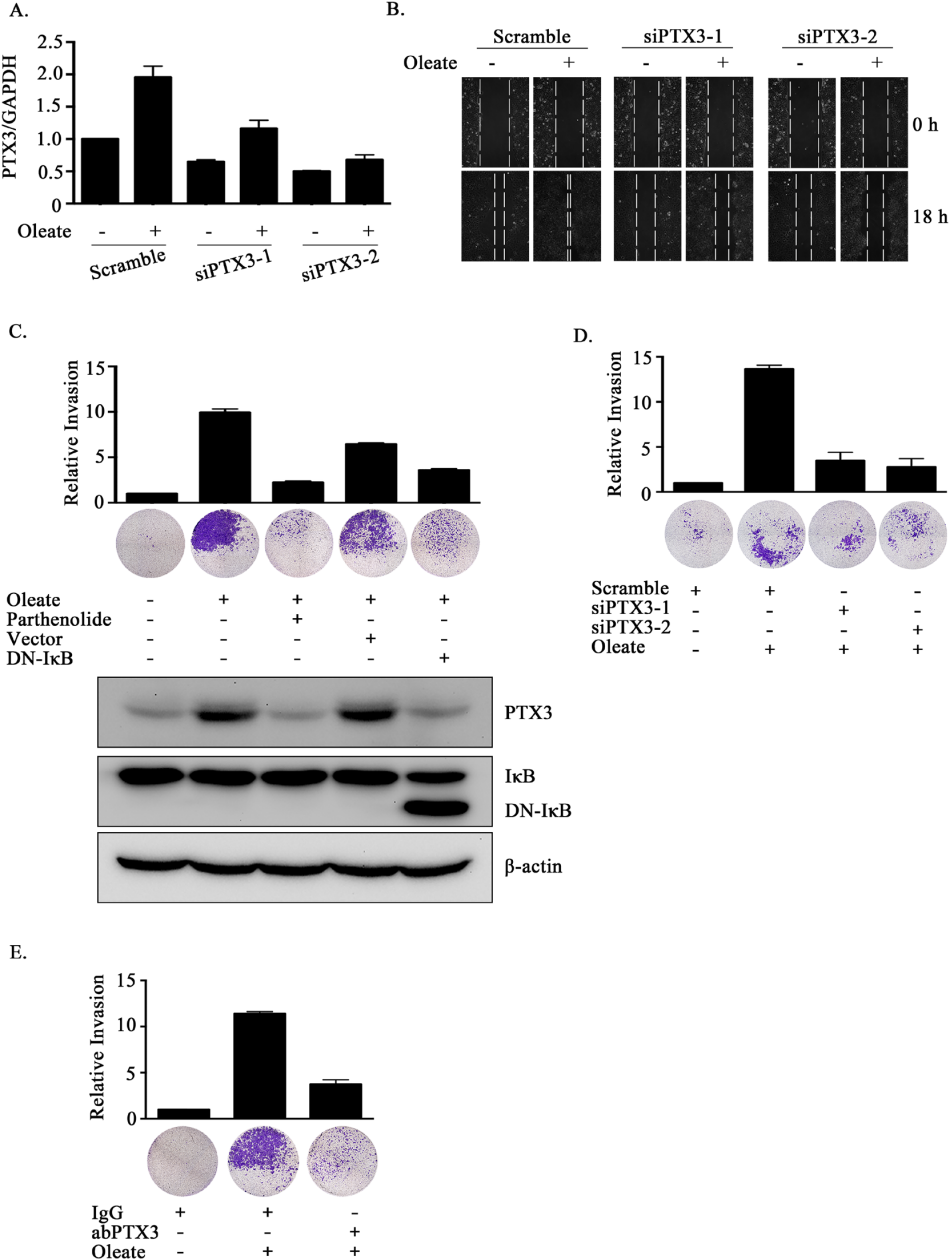
The depletion of PTX3 inhibits oleate-induced HNSCC migration and invasion **(A)** TU183 cells were transfected with 20 nM PTX3 siRNA (siPTX3) or scrambled oligonucleotides and treated with or without 400 μM oleate for 18 h. The mRNA expression levels of *PTX3* was normalized to the *GAPDH* mRNA level by real-time quantitative PCR. **(B and D)** TU183 cells were transfected with 20 nM PTX3 siRNA (siPTX3) or scrambled oligonucleotides by lipofection and then treated with 400 μM oleate for 18 h and 72 h for the migration and invasion assays, respectively. The wound-healing assay was performed as described in the “Materials and Methods” section. The migrating cells were examined using a microscope **(B)**. The invasive properties of the cells were examined using an invasion assay as described in the “Materials and Methods” section. The invading cells were fixed and stained with crystal violet and then examined using a microscope or the cells were solubilized with acetic acid, and the absorbance (OD, 595 nm) was measured in a microplate reader. The values are displayed the mean ± s.e.m. **(C-E)** TU183 cells were transfected with the DN-IκB expression vector by lipofection or treated with 10 μM parthenolide and then with 400 μM oleate **(C)**, immunoglobulin (IgG) or anti-PTX3 antibodies (1 μg/ml) **(E)**. The invasive properties of the cells were examined and measured. The values are the mean ± s.e.m.

**Figure 4:**
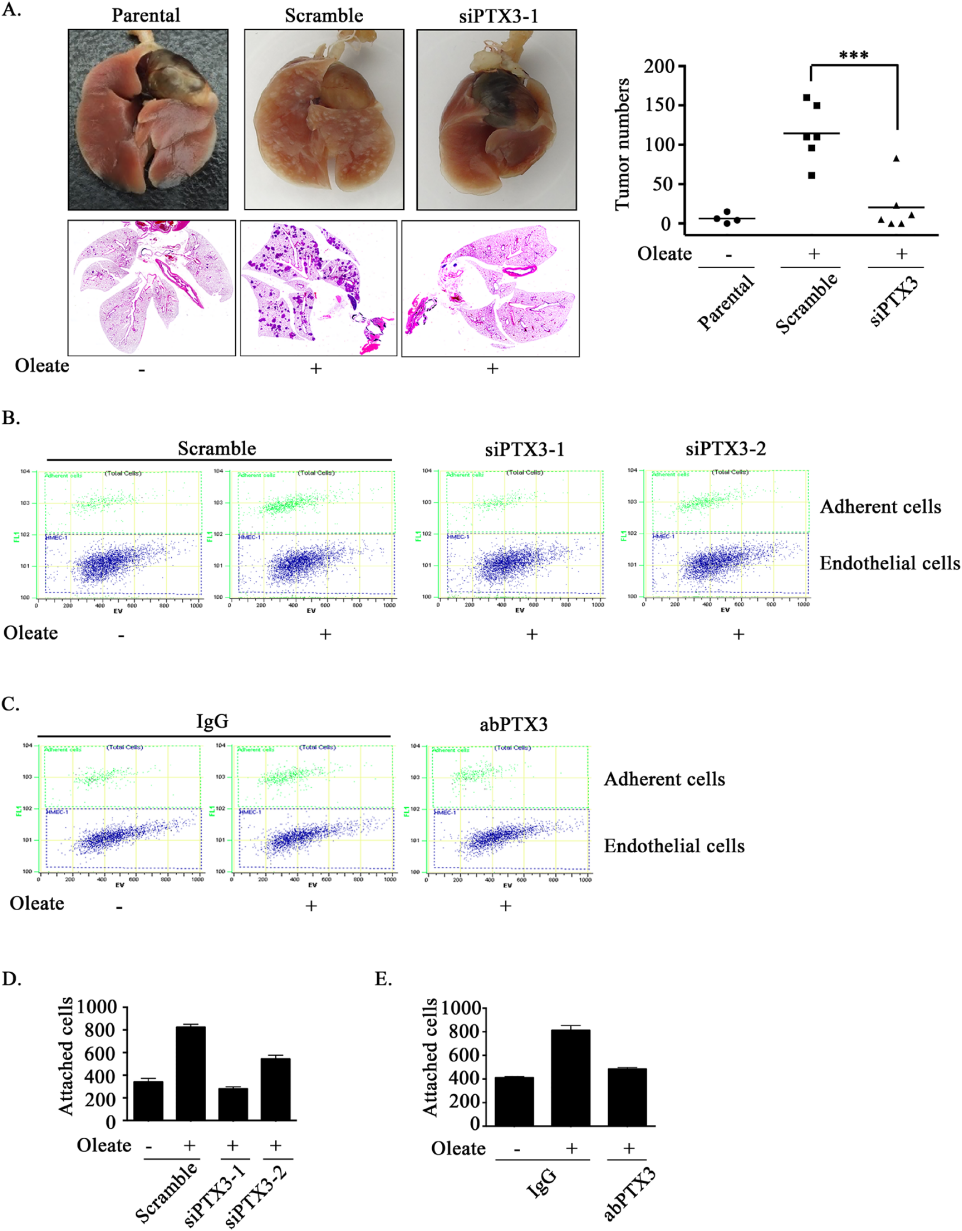
Oleate-induced autocrine production of PTX3 enhances tumor metastasis **(A)** TU183 cells were transfected with 20 nM PTX3 siRNA (siPTX3) or scrambled oligonucleotides by lipofection. A lung-colonization analysis was performed by injecting 1 × 10^6^ TU183 cells into the lateral tail vein of SCID mice. Prior to the injection, oleate was injected into the tail vein of mice to mimic the condition of patients who present with 400 μM circulating FFAs. Lung micronodules were examined and photographed after the mice were sacrificed at 6 weeks. The lungs and tumor tissues stained with H&E were examined under a microscope (left panel). The number of micronodules was counted under a microscope (right panel). Parental indicates TU183 cells, either with (N = 6) or without (N = 4) treatment with oleate. siPTX3 (siPTX3-1: N = 3, siPTX3-2: N = 3) indicates the knockdown of PTX3. The values represent the mean ± s.e.m. ***P <0.001. SC: scrambled oligonucleotides. **(B-E)** TU183 cells were transfected with 20 nM PTX3 siRNA oligonucleotides (siPTX3) and scrambled siRNA (SC) by lipofection, and the cells were treated with 400 μM oleate or anti-PTX3 antibodies (abPTX3) for 18 h. The cells were then labelled with CFSE and cultured with endothelial cells for 30 min. The bound tumor cells (adherent cells) were analyzed using a flow cytometer. TU183 cells were CFSE-positive, and endothelial cells were CFSE-negative. The bound tumor cells were quantified in three independent experiments by flow cytometry. The values are the mean ± s.e.m.

Lipid profiles are associated with breast cancer progression and invasion [35, 36]. However, the effects of oleate on HNSCC migration and invasion remain largely unclear. To elucidate the role of oleate-induced PTX3 in HNSCC migration and invasion, siRNA was used to deplete PTX3. Oleate-induced cell migration was significantly inhibited following the knockdown of PTX3 (Figure 3A and 3B). The oleate-induced invasion was eliminated following the inhibition of NF-κB signaling in parthenolide-treated cells and DN-IκB expressing cells (Figure 3C). In addition, the depletion of PTX3 dramatically blocked oleate-induced cell invasion (Figure 3D). The involvement of PTX3 in the regulation of oleate-induced invasion was also examined by disrupting the function of PTX3 with anti-PTX3 antibodies. The antibody neutralized PTX3 and significantly blocked oleate-induced cell invasion (Figure 3E). To determine the effects of PTX3 on oleate-induced metastasis *in vivo*, we investigated the distant dissemination (e.g., pulmonary colonization) of tumor cells using tail-vein injections in an animal model. Oleate was injected into the tail veins of mice to mimic the condition of patients who present with 400 μM circulating FFAs. Subsequently, parental or PTX3-depleted cells were injected into the tail vein. There was no significant lung injury observed in the mice treated with low-dose of oleate as shown in our previous studies [37]. As shown in Figure 4A, the circulating oleate induced tumor cell metastatic seeding of the lungs. However, the transient knockdown of PTX3 in tumor cells dramatically reduced the formation of pulmonary nodules induced by oleate (Figure 4A). On the other hand, to further clarify whether the reduction of metastatic nodules in PTX3-depleted cells was associated with the inhibition of tumor growth, tumor cell proliferation was examined in parental and siPTX3 cells. The cell proliferation and division rates remained unchanged between the parental and PTX3-knockdown cells (Supplementary Figure 2). The infiltration of tumor cells into distant locations through the circulatory system initially relies on their attachment to the blood vessels. Therefore, the ability of oleate-induced PTX3 to promote the interaction between the tumor cells and endothelial cells was examined. The enhancement of binding between tumor and endothelial cells was observed in oleate-treated parental but not PTX3-depleted cells (Figure 4B and 4D). In addition, the interaction was also blocked by the neutralization of PTX3 with anti-PTX3 antibodies (Figure 4C and 4E). These results indicate that the autocrine production of PTX3 via oleate induction stimulated the binding of tumor cells to endothelial cells, which enhanced pulmonary metastasis but not tumor growth.

### Oleate-induced PTX3 regulates HNSCC invasion through the induction of vimentin

**Figure 5:**
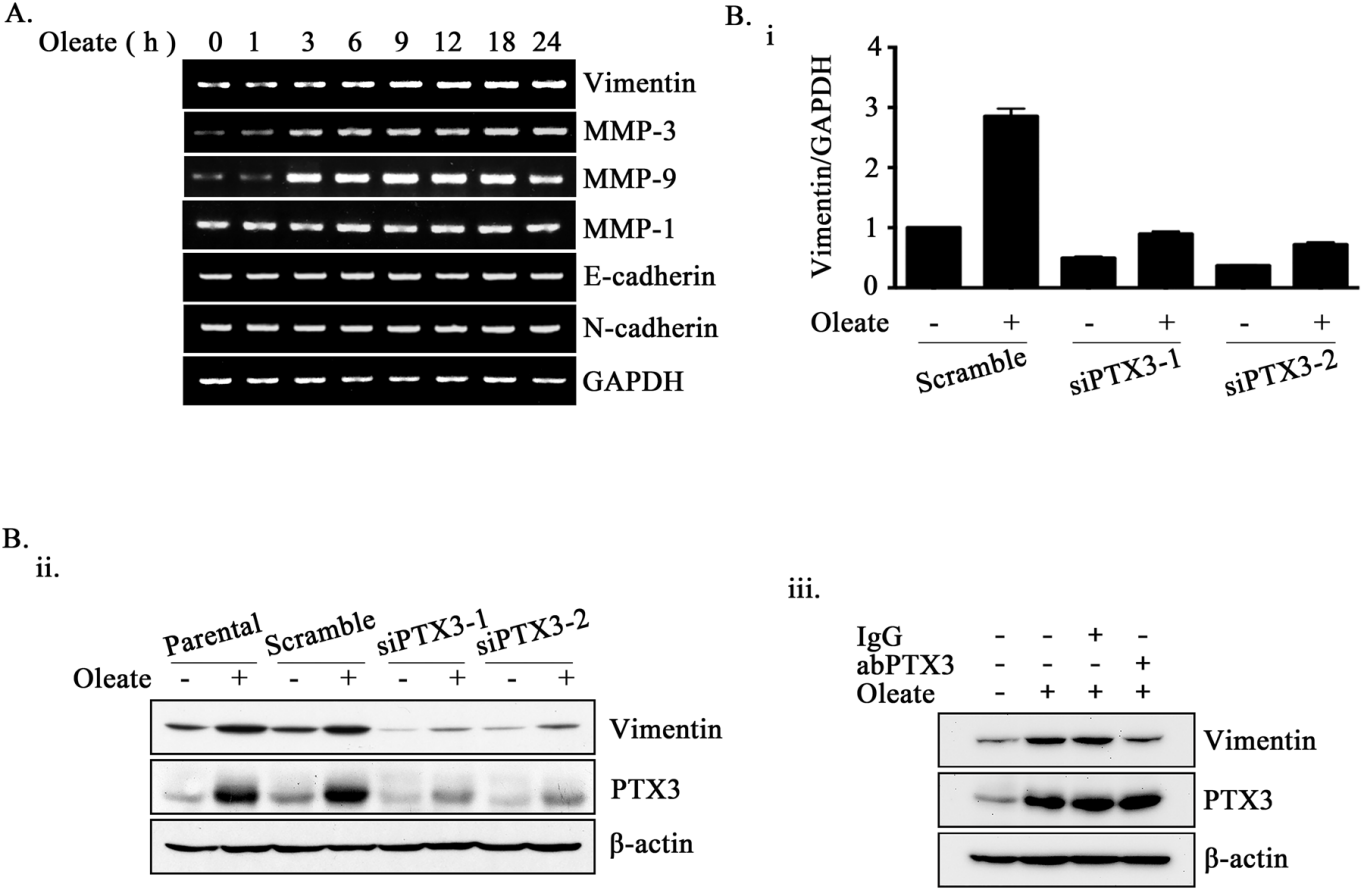
Oleate-induced PTX3 regulates the expression of vimentin **(A)** TU183 cells were treated with 400 μM oleate for the indicated period of time. The mRNA expression levels of EMT markers were examined using RT-PCR. **(B)** TU183 cells were transfected with 20 nM PTX3 siRNA (siPTX3) or scrambled oligonucleotides and treated with or without 400 μM oleate for 18 h. The mRNA expression levels of *vimentin* was normalized to the *GAPDH* mRNA level by real-time quantitative PCR (i). The values represent the mean ± s.e.m. of three determinations. Lysates of cells treated with oleate for 24 h or anti-PTX3 antibodies (abPTX3) (1 μg/ml) were prepared and subjected to SDS-PAGE and analyzed by Western blotting with antibodies against PTX3, vimentin and β-actin (ii and iii).

**Figure 6:**
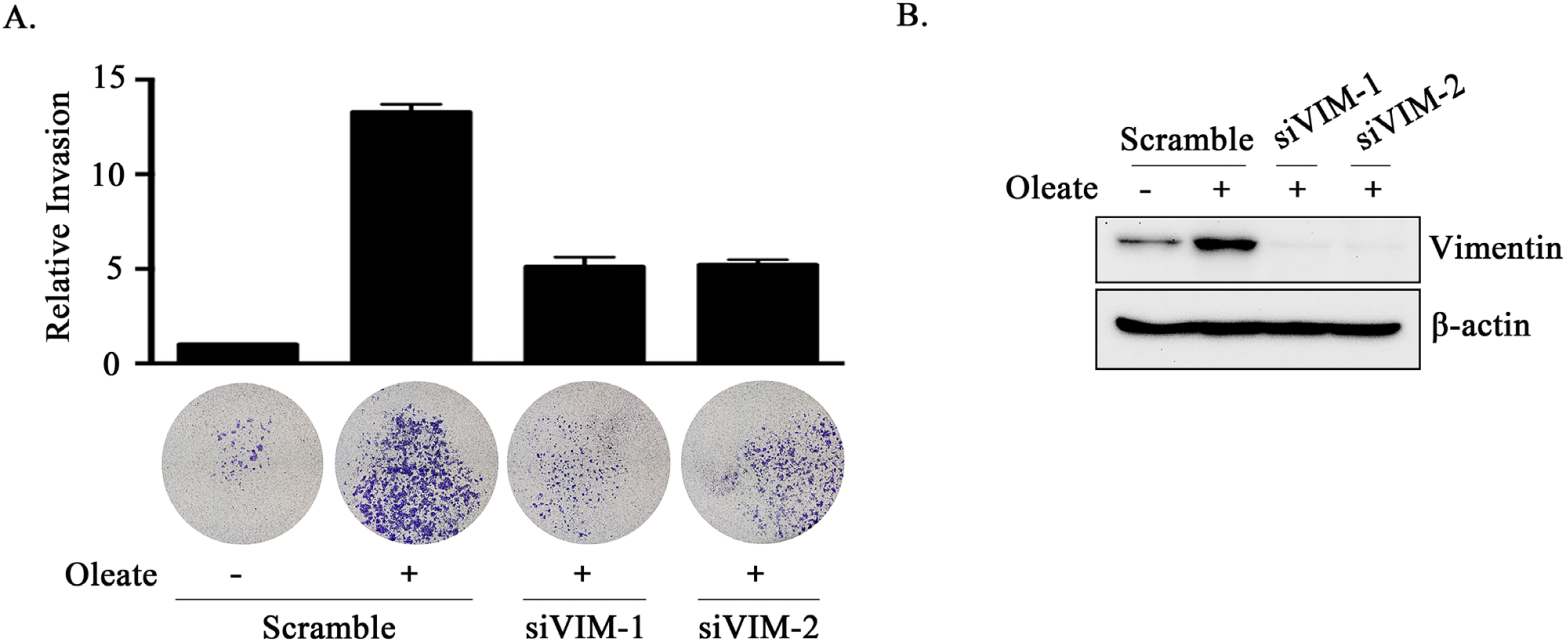
The knockdown of vimentin inhibits oleate-induced tumor cell invasion **(A and B)** TU183 cells were transfected with 20 nM vimentin siRNA (siVIM) or scrambled oligonucleotides by lipofection. The invasive properties of the cells were examined using an invasion assay as described in the “Materials and Methods” section. The invading cells were fixed and stained with crystal violet and then examined using a microscope or the cells were solubilized with acetic acid, and the absorbance (OD, 595 nm) was measured in a microplate reader **(A)**. The values are the mean ± s.e.m.. The expression of vimentin and β-actin was determined by Western blotting **(B)**.

Based on the observation that PTX3 expression was essential for oleate-enhanced cancer cell metastasis, we next studied the mechanisms involved in PTX3-regulated cell metastasis. Although no changes in N-cadherin, E-cadherin, or MMP-1 expression were observed in the oleate-treated cells, the expression levels of MMP-3, MMP-9 and vimentin were increased (Figure 5A). In addition, the depletion of PTX3 inhibited oleate-induced vimentin and MMP-3 but not MMP-9 expression (Figure 5B and Supplementary Figure 3). The neutralization of PTX3 using anti-PTX3 antibodies also blocked oleate-induced vimentin expression (Figure 5B). To further confirm the role of the oleate/PTX3/vimentin axis in tumor metastasis, the effects of vimentin knockdown on oleate-induced cell invasion were studied. The results showed that oleate-induced invasion was blocked in the vimentin-knockdown cells (Figure 6). We next investigated the association of the PTX3 and vimentin gene expression signature with HNSCC by data mining using the cancer microarray database Oncomine 4.0 (Oncomine DB at http://www.oncomine.org) [38]. The results demonstrated that PTX3 and vimentin expression was higher in malignant tissues than in normal tissues from HNSCC patients (Supplementary Figure 4). The results suggest that the oleate/PTX3/vimentin axis regulates HNSCC metastasis.

## DISCUSSION

Although the role of PTX3 in the regulation of the inflammatory response is clear, the function of PTX3 in the regulation of metastasis remains controversial. For example, PTX3 prevents fibroblast growth factor (FGF)-driven EMT to reduce the motility and invasive capacity of melanoma cells [39]. Moreover, PTX3 inhibits the FGF-induced mitogenic, angiogenic, and tumorigenic potential in steroid hormones-regulated tumors [40], prostate cancer cells [41] and breast cancer [42]. PTX3 acts as an extrinsic oncosuppressor by regulating complement-dependent tumor-promoting inflammation [43]. However, the expression of PTX3 promotes cervical cancer metastasis and EGF-induced HNSCC metastasis via the up-regulation of MMP-2 and MMP-9 [27, 29]. Therefore, to further clarify the function of PTX3 in HNSCC metastasis, we examined the effect of PTX3 on oleate-induced HNSCC metastasis. For the first time, we found that tumor-derived PTX3 promoted HNSCC metastasis but not tumor cell growth. In an *in vivo* assay, the metastatic seeding of the lungs by oleate-primed tumor cells was inhibited by the depletion of PTX3. Recent studies have also indicated that gastric cancer-derived PTX3 promotes tumor cell migration and macrophage recruitment, thus contributing to gastric cancer-related inflammation [44]. The elevated expression and secretion of PTX3 in breast cancer cells promote tumor bone-metastatic properties [45]. These results suggest that the tumor-derived PTX3 is essential for cancer metastasis.

According to our previous report [27] and this study, we found that EGF- and oleate-induced PTX3 promoted HNSCC metastasis through the induction of MMPs. The depletion of PTX3 inhibited EGF-induced MMP-9, which was consistent with the association between MMP-9 and PTX3 expression observed in cervical cancer cell metastasis [29]. Although oleate also induced MMP-9 and MMP-3 expression, only MMP-3 was regulated by oleate-induced PTX3, suggesting that the PTX3-regulated MMPs were dependent on specific types of stimuli, such as growth factors and fatty acids. In addition, the correlation between PTX3 and EMT markers such as vimentin remains unclear. In this study, we found that oleate-induced PTX3 enhanced vimentin expression to promote tumor invasion. In HNSCC patients, significantly higher MMP-3, MMP-9 and vimentin levels were found in metastatic tumors [46, 47]. Therefore, activation of the PTX3/MMPs and PTX3/vimentin signaling axes is a pathway involved in the regulation of EGF- and oleate-induced HNSCC metastasis.

In the regulation of PTX3 expression, several stimuli, such as IL-1β, tumor necrosis factor, lipopolysaccharides and EGF, have been identified [27, 48]. PTX3 promoter activity is strongly induced by these molecules through the activation of the NF-κB pathway [27, 49]. In this study, we further clarified that PTX3 was also induced in response to elevated oleate levels in cells. Although the activation of the NF-κB pathway was essential for oleate-induced PTX3 expression, oleate regulated PTX3 through the stabilization of mRNA and not by transcriptional regulation. These results suggested that the expression of PTX3 could be regulated by transcriptional and post-transcriptional modifications that are dependent on the stimuli. The activation of NF-κB also enhanced the expression of mRNA stabilizing factor HuR to promote gastric tumorigenesis [50], suggesting that the NF-κB-regulated mRNA stability may be through the HuR expression. In addition to NF-κB activation being essential for PTX3 expression, the importance of NF-κB activation is evident by the blocking of EMT elicited by indestructible IκB, an NF-κB inhibitor [51]. Furthermore, we found that oleate-induced PTX3 expression and tumor invasion were inhibited by DN-IκB and an NF-κB inhibitor. These results reveal that the induction of PTX3 by the oleate-activated NF-κB pathway contributes to NF-κB-promoted tumor metastasis. On the other hand, consistent with activation of PI-3K/ATK by oleate found in this study, the oleate was also via G-protein-coupled transmembrane receptor 40 (GPR40) to activate PI-3K/AKT, resulting in promoting breast cancer cell growth [52]. These results suggest that oleate enhanced tumorigenesis at least in part via the GPR40/PI-3K/AKT signaling pathway.

Upon metastasis, tumor cells must anchor to and interact with endothelial cells to successfully metastasize to distant sites. We found that oleate dramatically enhanced tumor-endothelial interactions by inducing the autocrine production of PTX3. Consistent with our studies, the elevation in vascular permeability by tissue injury after intestinal ischemia and reperfusion was dramatically repressed in PTX3 knockout mice [21]. These results indicate that PTX3 enhanced tumor metastasis not only by mediating vessel permeability but also by stimulating the adhesion of tumor cells to blood vessels. However, the mechanism involved in the PTX3-mediated stimulation of tumor-endothelial cell interaction and vessel permeability remains unknown. It is interesting that the regulation of tumor/endothelial interactions is demonstrated by the activation of the integrin β1 signaling pathway in breast cancer and HNSCC metastasis [53, 54]. Oleate also activated integrin β1 in HNSCC (our unpublished data), suggesting that the activation of integrin signaling might participate in PTX3-regulated tumor/endothelial interactions. In the recent report, extrinsically derived PTX3 acts as an oncosuppressor by regulating complement-dependent tumor-promoting inflammation [43]. On the other hand, for the first time, we found that the oleate-primed metastasis was regulated by the expression of PTX3 in tumor cells. The conflicting results raise the possibility that tumor-derived or extrinsically derived PTX3 play differential roles in the regulation of tumor progression. It may be due to the various production of PTX3 from different stages of tumor progression. For example, post translational modifications such as glycosylation might fine tune the functions of PTX3 in native immunity and inflammation [55]. Whether the roles of tumor-derived and extrinsically derived PTX3 in the regulation of metastasis and tumor growth, respectively, are associated with the glycosylation of proteins remains to be clarified.

In conclusion, we demonstrated the biological functions of PTX3 in oleate-induced HNSCC metastasis. Our results show that the oleate-induced autocrine production of PTX3 enhanced MMP-3 and vimentin expression and tumor/endothelial cell interactions to promote the metastatic seeding of tumor cells in the lungs. The measurement of PTX3 expression levels may provide prognostic information for the treatment of HNSCC metastasis, which suggests that PTX3 could be a novel therapeutic target for dyslipidemia-regulated HNSCC metastasis.

## MATERIALS AND METHODS

### Cell culture

The mouth epidermoid carcinoma cell line (KB) and pharynx squamous cell carcinoma cell line (FaDu) were purchased from the American Type Culture Collection (ATCC, Manassas, VA, USA). The nasopharyngeal carcinoma cell line (HONE1) and oral cancer cell line (TU183) were kindly provided by Dr. Kwang-Yu Chang (National Health Research Institutes, Taiwan). The human microvascular endothelial cell line (HMEC-1) was kindly provided by Dr. Trai- Ming Yeh (Department of Medical Laboratory Science and Biotechnology, Medical College, National Cheng Kung University). The cell line of human head and neck cancer cells (TU183) was grown at 37°C under 5% CO_2_ in 10 cm plastic dishes containing 10 ml of Dulbecco's modified Eagle's medium supplemented with 10% fetal bovine serum, 100 μg/ml streptomycin, and 100 units/ml penicillin. The cell lines of human head and neck cancer cells, KB, HONE1 and FaDu were also grown in same manner but with different medium, including RPMI (KB and HONE1) and Minimum Essential Media (MEM, for FaDu cells) Medium. The human microvascular endothelial cell line (HMEC-1) was also grown in same manner but with MCDB 131 medium supplemented with 50 μg/ml Endothelial Cell Growth Supplement ( ECGS, Millipore, Billerica, MA).

### Preparation of fatty acid/albumin complexes

Fatty acid/albumin complexes were prepared according to the method previously described [56]. Briefly, the sodium salt of oleate, palmitate and linoleic acid (Sigma-Aldrich, St Louis, MO, USA) was dissolved in water to give a final concentration of 20 mM. Bovine serum albumin (BSA, fatty acid free) (US Biological, Swampscott, MA, USA) was dissolved in calcium and magnesium free PBS (Sigma-Aldrich, St Louis, MO, USA). 3.5 ml of the warmed 20 mM sodium oleate, palmitate and linoleic acid solution was dropwise added to 11.6 ml of warmed 5 % BSA to form fatty acid/albumin complexes in an 8:1 molar ratio. The fatty acid/albumin complexes were added to 160 ml warmed culture medium to achieve culture medium containing 400 μM oleate, palmitate and linoleic acid. The medium was freshly prepared upon performing experiments and diluted to desired concentration of oleate, palmitate and linoleic acid if experiments needed.

### Western blotting

An analytical 12% SDS-PAGE was performed, and 20 μg of protein from each fraction was analyzed unless stated otherwise. Western blotting was performed as previously described [57]. Antibodies against human phospho-AktS473, Vimentin, phospho-IκBαS32/36 phospho-ERK1/2T202/Y204 (Cell Signaling Technology, Danvers, MA), PTX3, IκBα, ERK1/2 (Santa Cruz Biotechnology, Inc., Santa Cruz, CA) and β-actin (Sigma, St. Louis, MO) were used as the primary antibodies.

### Separation of cytoplasmic and nuclear fraction

TU183 cells were treated with 400 μM oleate at indicated time points. The cytoplasmic fractions and nuclear extracts of cells were prepared for Western blotting analysis according to the method previously described [58]. Briefly, the cytoplasmic protein of 5*10^6^ TU183 cells were harvested by scraping in cold phosphate-buffered saline (PBS), lysed in 400 μl cold buffer A (10 mM Hepes, pH 7.9, 1.5 mM MgCl_2_, 10 mM KCl, 0.5 mM DTT, 0.5 mM PMSF, 2 μg/ml leupeptin, 2 μg/ml pepstatin, 1 mM Sodium vanadate) by 12 passages through a 25-gauge needle. After centrifugation for 10 min at 1000 g at 4 °C, the nuclear pellet was resuspended in 400 μl cold buffer C (20 mM Hepes, pH 7.9, 1.5 mM MgCl_2_, 420 mM NaCl, 0.2 mM EDTA, 25 % glycerol, 0.5 mM DTT, 0.5 mM PMSF, 2 μg/ml leupeptin, 2 μg/ml pepstatin, 1 mM Sodium vanadate) and incubated on ice for 20 min. The nuclear extract was centrifuged for 10 min at 12,000 g at 4 °C. The supernatant was nucler protein, divided into aliquots, and stored at -70 °C. For detecting the ratios of nuclear and cytoplasmic parts of NF-κB, equal volumes of nuclear and cytoplasmic lysate were subject to western blotting analyses with anti-NF-κB p65 (Santa Cruz Biotechnology, Inc., Santa Cruz, CA), HDAC-1 (Millipore, Billerica, MA) and α-Tubulin antibodies.

### Reverse transcription-PCR and real-time quantitative RT-PCR

Total RNA was isolated using the TRIzol RNA extraction kit (Invitrogen), and 2 μg of RNA was subjected to reverse transcription-PCR using a reverse transcriptase reaction kit (Invitrogen Life Technologies, Carlsbad, CA) according to the manufacturer's instructions. The *PTX3*-specific primers (sense, 5′-CATCCAGTGAGACCAATGAG-3′; antisense, 5′-GTAGCCGCCAGTTCACCATT-3′), *MMP1*-specific primers (sense, 5′-ATGCACAGCTTTCCTCCACT-3′; antisense, 5′-TTCCCAGTCACTTTCAGCCC-3′), *MMP3*-specific primers (sense, 5′-GCAAGACAGCAAGGCATAGAG-3′; antisense, 5′-CCGTCACCTCCAATCCAAGG-3′), *MMP9*-specific primers (sense, 5′-GACAAGAGCCAGGAAGAAACC-3′; antisense, 5′-CTTTAGCACTCCTTGGCAAAAC -3′), *Vimentin*-specific primers (sense, 5′- TGGCCGACGCCATCAACACC -3′; antisense, 5′- CACCTCGACGCGGGCTTTGT -3′), *E-cadherin*-specific primers (sense, 5′- TCCATTTCTTGGTCTACGCC -3′; antisense, 5′- CACCTTCAGCCAACCTGTTT -3′), *N-cadherin*-specific primers (sense, 5′- GTGCCATTAGCCAAGGGAATTCAGC -3′; antisense, 5′- GCGTTCCTGTTCCA CTCATAGGAGG -3′), and glyceraldehyde-3-phosphate dehydrogenase-specific primers (sense, 5′-CCATCACCATCTTCCAGGAG-3′; antisense, 5′-CCTGCTTCACCACCTTCTTG-3′) were used. The PCR products were separated by 2% agarose gel electrophoresis and visualized with ethidium bromide staining. For the quantitative real-time RT-PCR, cDNA synthesis was performed using the TITANIUM One-Step RT-PCR kit (Clontech) containing SYBR Green I. Real-time fluorescence monitoring and the melting curve analysis were performed with the LightCycler according to the manufacturer's recommendations. The relative transcript levels of the target genes were calculated using the standard curves obtained by serial RNA dilutions and were normalized to the transcript levels of glyceraldehyde-3-phosphate dehydrogenase (GAPDH) in the same RNA sample.

### Enzyme-linked immunosorbent assay

Quantitation of the secretion of PTX3 in the culture medium was achieved by an enzyme-linked immunosorbent assay (ELISA) according to the manufacturer's instructions (R&D Systems). Briefly, after oleate treatment, 100 μl of the culture medium was collected and incubated with precoated capture antibodies at room temperature in a 96-well microplate. After a washing step, 100 μl of detection antibody was added and incubated for another 2 h at room temperature. Next, 100 μl of a working dilution of Streptavidin-HRP was added into each well and incubated for 20 min under protection from light. After a washing step, 100 μl of substrate solution was added and incubated for 20 min under protection from light. After adding 50 μl of stop solution to each well, the plate was gently tapped to ensure thorough mixing, and a microplate reader was used to quantify the optical density at 450 nm immediately.

### Plasmid construction

Dominant negative IκB mutant was generated by N-terminal deletion of residues 1-45 using a standard PCR approach [33]. The DNA fragment bearing the promoter region of PTX3 (1200 bp) was constructed as previously described [27].

### DNA transfection and luciferase assay

The transient transfection of cells with plasmids was performed with Lipofectamine 2000 (Invitrogen) according to the manufacturer's instructions but with slight modification. Cells were replated 24 h before transfection at a density of 3 × 10^5^ cells in 2 ml of fresh culture medium in a 3.5-cm plastic dish. For the transfection, 2 μl of Lipofectamine 2000 was incubated with 0.5 μg of plasmid in 1 ml of Opti-MEM medium for 30 min at room temperature. Cells were transfected by replacing the medium with 1 ml of Opti-MEM medium containing the plasmids and Lipofectamine 2000, and then, the cells were incubated at 37 °C in a humidified atmosphere of 5% CO_2_ for 24 h. The luciferase activity in the cell lysate was determined as described previously [57].

### Immunofluorescence

Cells were grown on chamber slides and fixed with 4% paraformaldehyde (Sigma Corporation, Cream Ridge, NJ, USA) in phosphate-buffered saline at 25 °C for 30 min. The cells were then rinsed with phosphate-buffered saline three times, permeabilized, and blocked with 1% Triton X-100 and 2% FBS for 15 min. Next, the cells were incubated with NF- κB p65 antibodies (Santa Cruz Biotechnology, Inc., Santa Cruz, CA) at a dilution of 1:100 for 1 h and treated with Alexa Fluor® 488 goat anti-rabbit IgG polyclonal antibodies at a dilution of 1:500 for 30 min. Finally, the cells were counterstained with 0.2 μg/μl 4, 6-diamidino-2-phenylindole (Invitrogen, Life technologies, Carlsbad, CA, USA) and mounted using a 90% glycerol mounting solution. The digital images were acquired using an Olympus BX51 microscope (Olympus America, Inc., Melville, NY, USA).

### Transfection with siRNA oligonucleotides

The transient transfection of cells with 20 nM PTX3 (siRNA IDs: HSS108892, HSS108893) or Vimentin (siRNA IDs: HSS111287, HSS187671) siRNA oligonucleotides was performed using RNAiMAX (Invitrogen, Grand Island, NY) according to the manufacturer's instructions with slight modifications. For the transfection, 6 μl of RNAiMAX was incubated with PTX3/Vimentin siRNA or scrambled siRNA (Invitrogen, Grand Island, NY) in 200 μl of Opti-MEM medium for 5 min at room temperature. Cells were transfected by replacing the culture medium with medium containing the siRNA and RNAiMAX, and then, the cells were incubated at 37 °C in a humidified atmosphere of 5% CO_2_ for 24 h. After incubation, the medium was replaced with fresh culture medium to conduct subsequent experiments.

### Wound healing assay

For the wound healing assay, 3.5 × 10^4^ cells were seeded in 12-wells containing the linear spacer inserts. Following overnight culture, the linear spacer inserts were removed, which created a regular and defined “wound” within the cell monolayer. After phosphate-buffered saline wash, the wells were either left untreated or treated with oleate in medium containing 2% FBS unless stated otherwise. The extent of wound closure was observed using the phase-contrast microscope camera Olympus BX51 (Olympus America, Inc., Melville, NY, USA).

### Cell adhesion assay

Briefly, the tumor cells were treated with 400 μM oleate and/or 1 μg/ml PTX3 antibodies for 18 h, then labeled for 30 min at 37 °C with Carboxyfluorescein succinimidyl ester (CFSE) (Invitrogen Life Technologies, Carlsbad, CA), and washed twice with phosphate-buffered saline. The tumor cells (3 × 105 cells/ml) were resuspended and added to a monolayer of HMEC-1 cells. After incubation for 30 min at 37 °C, the wells were gently washed twice with phosphate-buffered saline to remove the non-adherent cells. The number of adherent tumor cells and HMEC-1 cells was quantified by the Cell Lab Quanta SC flow cytometer (Beckman Coulter, Fullerton, CA, USA) equipped with an argon laser. The CFSE emission was measured at 525 nm (FL1). The bound tumor cells were determined by the ratio of the bound tumor cells (adherent cells) that were CFSE-positive to the HMEC-1 cells that were CFSE-negative (3 × 103 cells per group).

### Transwell invasion assay

The assays were performed using Millicell™ hanging cell culture inserts (polyethylene terephthalate (PET) membranes with 8 μm pores) (Millipore). A total of 4 × 104 cells were plated in medium containing 2% serum in the upper chamber on a 10% Matrigel-coated membrane, while the lower chamber was filled with medium containing 2% serum unless stated otherwise. After incubation for 72 h, the cells in the upper chamber were removed and the invaded cells at the bottom of the membrane were fixed with 4% Paraformaldehyde (PFA). The invaded cells were then treated with methanol and stained with 0.5% crystal violet. To quantify the invaded cells, the crystal violet stained cells were washed with water and then lysed with 10% acetic acid. The absorbance of the lysates was measured at 595 nm. The OD values of the invaded cells are shown in the graphs. The quantification was determined from three independent experiments.

### Tumor metastasis assay in an animal model

Tumor metastasis was initiated by an intravenous (tail vein) injection of cancer cells into 6-week-old male severe combined-immunodeficient (SCID) mice. There was no randomization method or blinding group for the allocation of the animals. Briefly, oleate was injected into the circulation of mice to mimic the condition of patients who present with 400 μM circulating FFAs. The animals were then injected with 1 × 106 cells mixed in phosphate-buffered saline, and all of the mice were sacrificed at six weeks after the injection using an ethical method. The mice were obtained from the National Cheng Kung University Laboratory Animal Center (Tainan, Taiwan) and the National Laboratory Animal Center (Tainan, Taiwan). The animal study was approved (Approval No. NCKU-IACUC-102-078) by the Laboratory Animal Committee of the National Cheng Kung University. Hematoxylin and eosin staining (H&E) was performed by the Human Biobank at the Research Center of Clinical Medicine of the National Cheng Kung University Hospital.

### Statistical analyses

Statistical analyses were performed using GraphPad Prism 6.0 (La Jolla, CA, USA). Data are shown as the mean ± s.e.m. of three independent experiments. Center values indicate the mean. A P-value less than 0.05 was considered significant and was denoted by *. A P-value less than 0.01 and 0.001 was denoted by ** and ***, respectively.

## SUPPLEMENTARY MATERIALS FIGURES


