# Supplementary material for: Oleate-induced PTX3 promotes head and neck squamous cell carcinoma metastasis through the up-regulation of vimentin
Source: Oncotarget. 2017 Apr 21;8(25):41364–78. doi: 10.18632/oncotarget.17326 (PMC5522334; doi:10.18632/oncotarget.17326)
Supplement: Supplementary file 1 [file oncotarget-08-41364-s001.pdf]

Oleate-induced PTX3 promotes head and neck squamous cell carcinoma metastasis through the up-regulation of vimentin

SUPPLEMENTARY MATERIALS

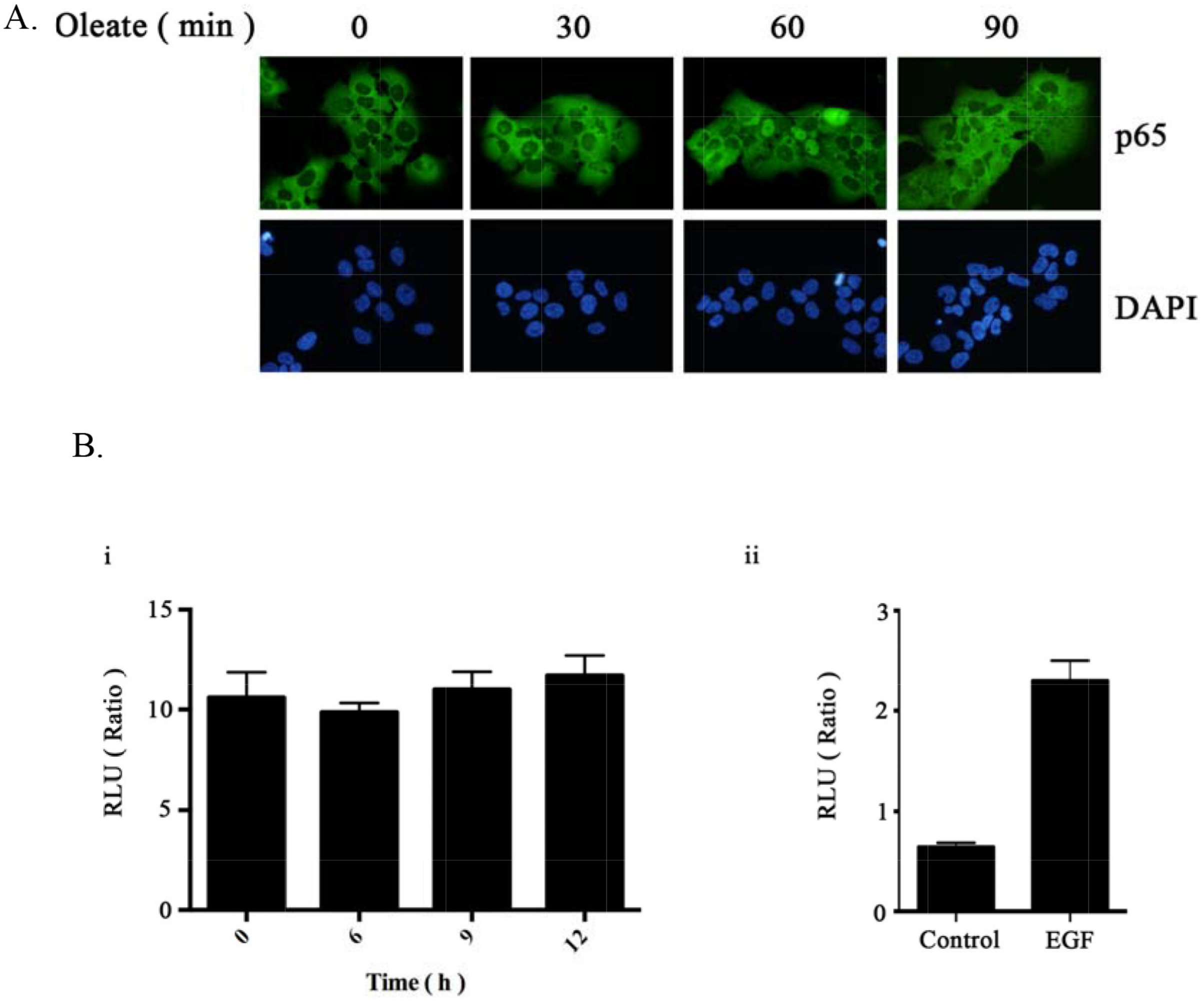

Supplementary Figure 1: Oleate treatment induces the translocation of NF-κB but does not affect the PTX3 promoter activity. (A) Cells were fixed using 4% paraformaldehyde, labeled with anti-p65 antibodies, and then stained with secondary antibodies conjugated with fluorescein isothiocyanate. DNA was stained with 4, 6-diamidino-2-phenylindole (DAPI). Finally, the cells were examined using a microscope. **(B)** TU183 cells were co-transfected with 0.5 µg of the PTX3 promoter and 20 ng of the renilla luciferase construct by lipofection. The cells were treated with 400 µM oleate for the indicated period of time (i) or 50 ng/ml EGF for 6 h (ii). The firefly and renilla luciferase activities were then determined and normalized. The values are the mean ± s.e.m.

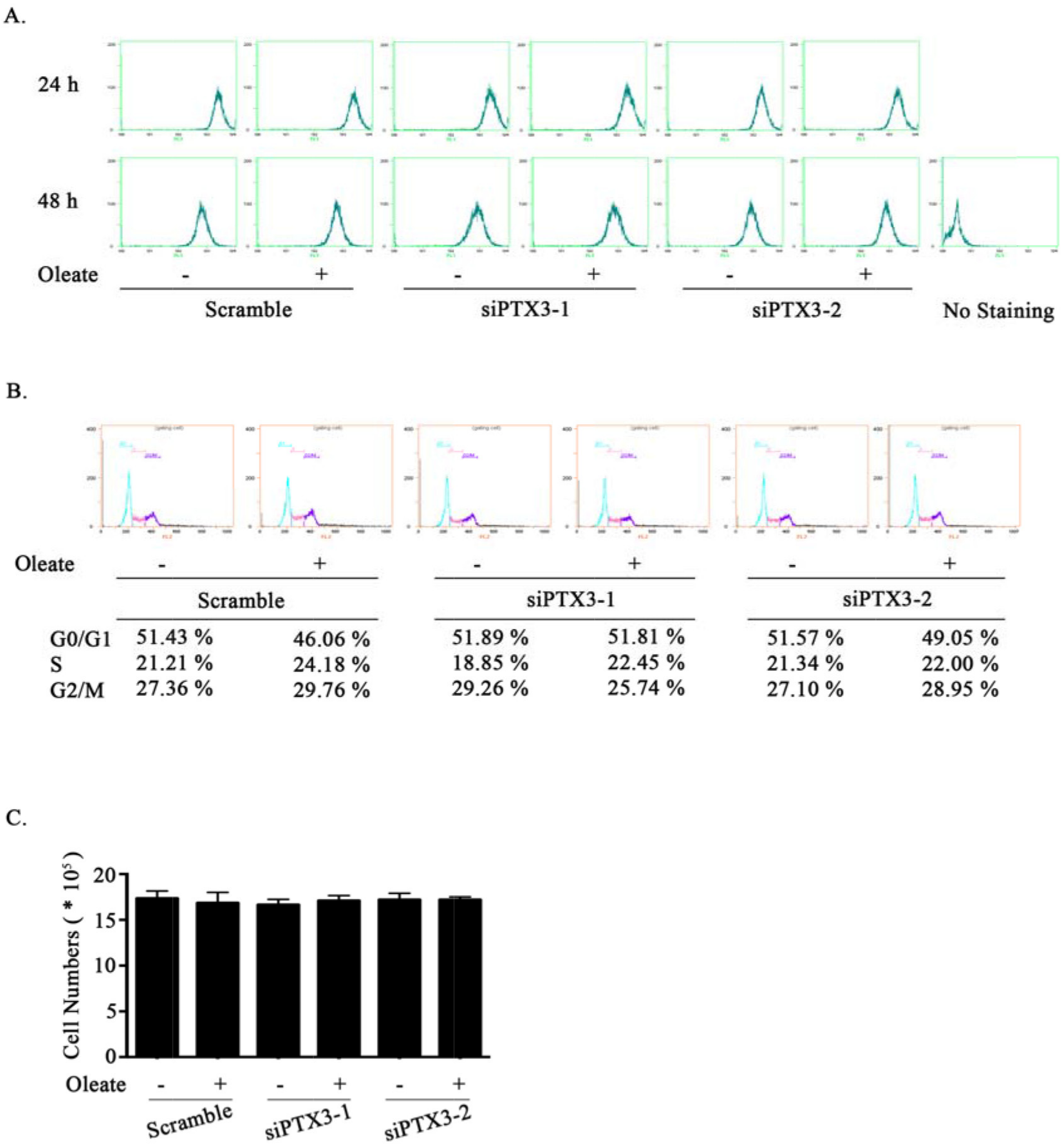

Supplementary Figure 2: Oleate-induced PTX3 has no effect on tumor cell proliferation. **(A-C)** TU183 cells were transfected with 20 nM PTX3 siRNA (siPTX3) or scrambled oligonucleotides by lipofection and treated with or without 400 µM oleate for 24~48 h. The cell proliferation assay was performed by CFSE staining, and the quantification was achieved by flow cytometry (Beckman Coulter). CFSE emission was measured at 585 nm **(A)**. The cell-cycle analysis was performed by propidium iodide staining. The cell cycle phase gates were drawn using the approximations made by the Dean-Jett-Fox cell cycle modeling algorithm **(B)**. The cells were stained with trypan blue, and the number of cells was counted **(C)**. The values are the mean ± s.e.m.

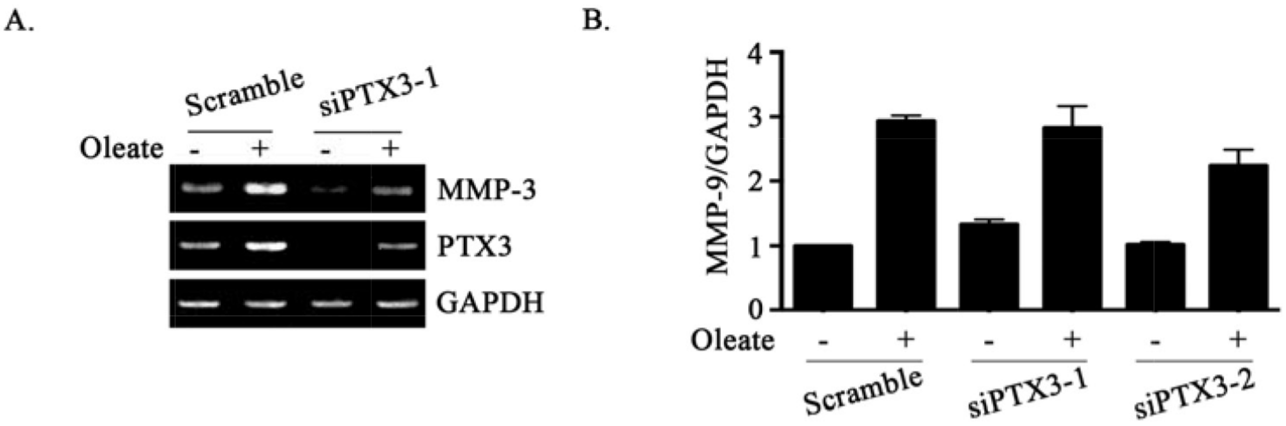

Supplementary Figure 3: The depletion of PTX3 inhibited oleate-induced MMP-3 expression but not MMP-9 expression. (A and B) TU183 cells were transfected with 20 nM PTX3 siRNA (siPTX3-1) or scrambled oligonucleotides by lipofection and treated with or without 400 μM oleate for 6 h. The mRNA expression of MMP-3 and MMP-9 were analyzed by RT-PCR **(A)** and quantitative PCR **(B)**. The values are the mean ± s.e.m.

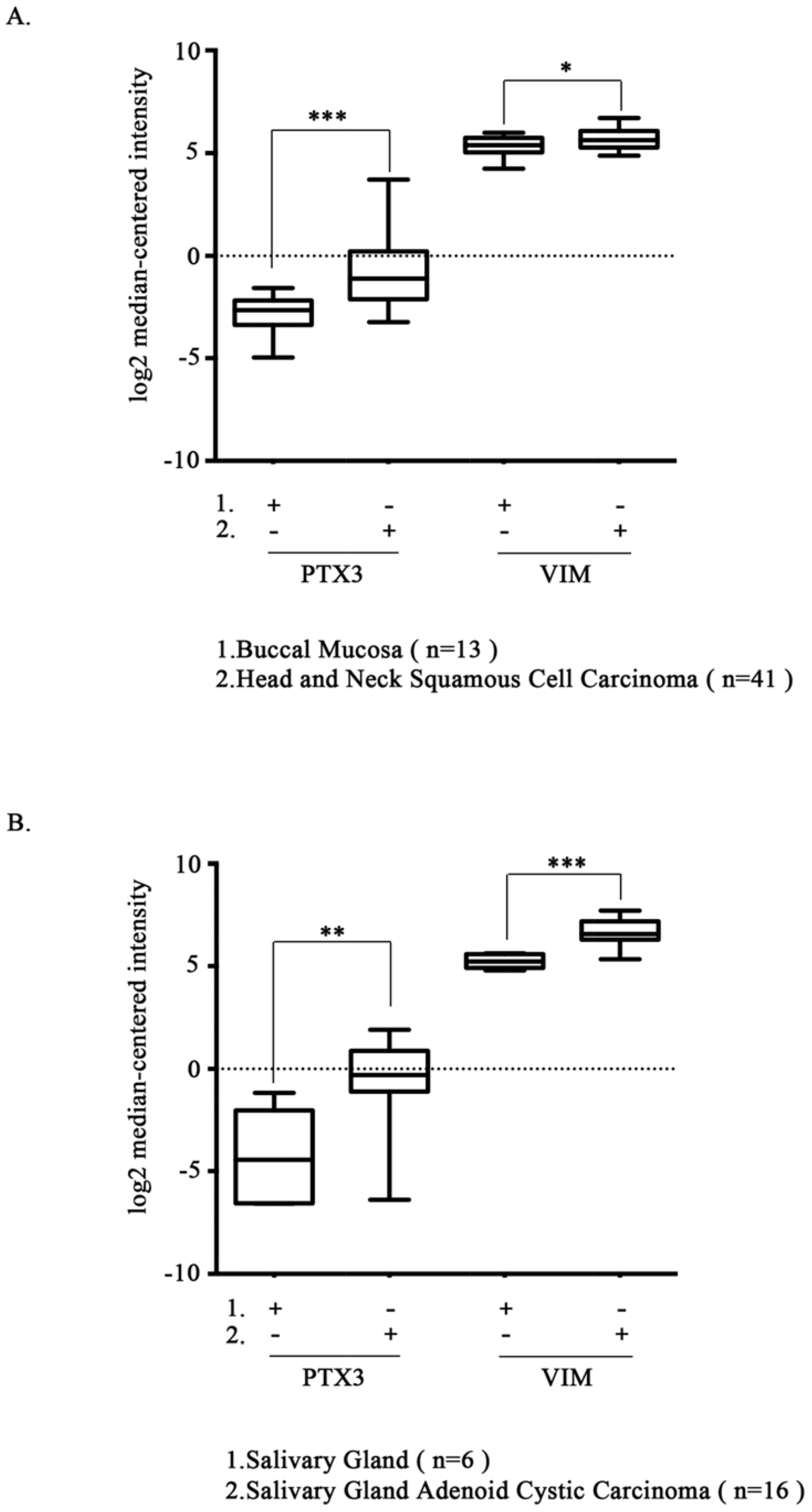

Supplementary Figure 4: Up-regulation of PTX3 and vimentin in HNSCC. **(A and B)** Data mining was performed on the cancer microarray database Oncomine 4.0 (Oncomine DB at www.oncomine.org). Oncomine box plot of *PTX3 and vimentin* expression levels between human normal tissues and head and neck cancer in multiple datasets from refs. 1 **(A)** and 2 **(B)**. The values are indicated as the mean + s.e.m. *: $P<0.05$; **: $P<0.01$; ***: $P<0.005$. Ref 1: Ginos MA, et al. Cancer Res 2004;64:55-63. Ref 2: Frierson HF Jr, et al. Am J Pathol 2002;161:1315-23.
